# Effect of the interplay between protein and surface on the properties of adsorbed protein layers

**DOI:** 10.1016/j.biomaterials.2014.04.012

**Published:** 2014-08

**Authors:** Myriam M. Ouberai, Kairuo Xu, Mark E. Welland

**Affiliations:** Nanoscience Centre, Department of Engineering, University of Cambridge, Cambridge CB3 0FF, UK

**Keywords:** Protein adsorption, DPI, QCM-D, Layer properties, Percentage solvation, Viscoelastic property

## Abstract

Although protein adsorption to surface is a common phenomenon, investigation of the process is challenging due to the complexity of the interplay between external factors, protein and surface properties. Therefore experimental approaches have to measure the properties of adsorbed protein layers with high accuracy in order to achieve a comprehensive description of the process. To this end, we used a combination of two biosensing techniques, dual polarization interferometry and quartz crystal microbalance with dissipation. From this, we are able to extract surface coverage values, layer structural parameters, water content and viscoelastic properties to examine the properties of protein layers formed at the liquid/solid interface. Layer parameters were examined upon adsorption of proteins of varying size and structural properties, on surfaces with opposite polarity. We show that “soft” proteins such as unfolded α-synuclein and high molecular weight albumin are highly influenced by the surface polarity, as they form a highly diffuse and hydrated layer on the hydrophilic silica surface as opposed to the denser, less hydrated layer formed on a hydrophobic methylated surface. These layer properties are a result of different orientations and packing of the proteins. By contrast, lysozyme is barely influenced by the surface polarity due to its intrinsic structural stability. Interestingly, we show that for a similar molecular weight, the unfolded α-synuclein forms a layer with the highest percentage of solvation not related to surface coverage but resulting from the highest water content trapped within the protein. Together, these data reveal a trend in layer properties highlighting the importance of the interplay between protein and surface for the design of biomaterials.

## 1 Introduction

Knowledge of protein adsorption has expanded rapidly in the past decades due to the development of techniques and methods providing more detailed experimental data [1]. The range of techniques used to characterize protein layers includes optical techniques such as ellipsometry [2,3], neutron reflectometry [4], surface plasmon resonance [5], waveguide lightmode spectroscopy [6], and dual polarisation interferometry (DPI) [7,8]; acoustic biosensing techniques such as quartz crystal microbalance with dissipation (QCM-D) [9,10]; surface imaging techniques such as atomic force microscopy [11,12] and finally techniques focusing on the secondary structure of the adsorbed protein such as attenuated total reflectance infrared spectroscopy [13]. These techniques measure the kinetics of protein adsorption, mass coverage and structure of the layer, thus achieving a description of the adsorption process. In addition to this, a combination of these techniques has been used to characterize specific protein layers in more details [14–16].

Apart from the external parameters, such as temperature [17], pH [18], and ionic strength [19], other critical fixed parameters influence the properties of the resulting layers; these include both protein and surface properties [1]. The properties of the adsorbed layer are highly dependent on the size, net charge and structure of constituent proteins as these factors influence surface affinity, protein packing and orientation and water content of the layer. Furthermore, the polarity [13,20] and roughness [21] of the surface influence the protein-surface interaction in terms of protein affinity, reversibility of the adsorption process and the extent of protein deformation. Therefore the complexity of the interplay of these parameters on the properties of protein layers requires a highly accurate experimental approach in order to achieve systematic model descriptions [1].

Our previous work described in details the properties of adsorbed lysozyme layers as function of surface coverage, using a combination of QCM-D and DPI [16]. The use of these two techniques has proved to be valuable in determining structure–property relationships between surface coverage and adsorbed lysozyme properties on a hydrophilic silica substrate. In particular, we show that solvent content, layer rigidity and protein orientation and packing are dependent of the surface coverage. These insights emerge from the complementary data extracted from the two techniques, such as ‘dry’ and ‘wet’ mass values, layer solvation, thickness, density and viscoelastic properties.

In the present study, we use the same approach to investigate the layer properties of three proteins of different size and structure, adsorbed onto two surfaces of opposite polarity. A comparison of the layer properties is made between alpha-synuclein (α-Syn) and lysozyme, which are of similar molecular weight (∼14 kDa) but contrasting structure: highly ordered in the case of lysozyme and unfolded in the case of α-Syn. Further comparison is made with bovine serum albumin (BSA), a globular protein with a higher molecular weight (∼66 kDa). These proteins are adsorbed onto a hydrophilic, negatively charged silica substrate and onto a hydrophobic methylated substrate. The effects of surface polarity on the proteins' affinity and their resulting packing and orientation upon adsorption are investigated. Overall, this study aims to draw some conclusions about the influence of the interplay between the protein and surface on the properties of the resulting adsorbed protein layers.

## 2 Materials and methods

### 2.1 Materials

Human milk lysozyme (14.4 kDa), bovine serum albumin (BSA, 66.4 kDa), HPLC Grade water (resistivity >18 mΩ cm), monobasic and dibasic phosphate, sodium deodecyl sulphate (SDS), trichloro(methyl)silane, and toluene (anhydrous, 99.8%) were purchased from Sigma (Sigma & Aldrich, UK) and used as received. α-synuclein (α-Syn, 14.46 kDa) was prepared as previously described [22]. Millex syringe filter (pore size = 0.22 μm) was obtained from Fisher (Fisher Scientific, UK). Diluted Hellmanex^®^ III (Hellma Analytics, Germany) solution (2%, in deionised water) was used to clean the DPI injection loops.

Phosphate solution (10 mm, pH 7.4) used as buffer solution was prepared using monobasic and dibasic phosphate and HPLC grade water. Filtered protein stock solutions were each prepared in the same buffer solution and then diluted for injection at the desired concentrations. The concentrations of diluted protein solutions were checked by UV–Vis spectroscopy (Varian Cary^®^ 300 UV–Vis Spectrophotometer, Agilent Technologies, UK) at 280 nm. Untreated silicon oxynitride (Farfield–Biolin Scientific AB, Sweden) and silicon dioxide (Q-Sense-Biolin Scientific AB, Sweden) sensor chips used for the DPI and QCM-D experiments respectively, were cleaned prior to the experiments and were referred to as the ‘hydrophilic surface’ and ‘Silicon oxide’ surface in later sections (for detailed cleaning procedure, please refer to reference [16]). Silanization of the cleaned sensor chips was performed in the following way: toluene solution immersion with agitation for 30 s, followed by immersion of toluene with 4% trichloro(methyl)silane solution for 1 h. The chips were then blown dried with nitrogen gas. The silane-modified chips are referred to as the ‘hydrophobic surface’ and ‘Methyl’ surface in later sections. Contact angle measurements were performed to check the polarity of the hydrophilic and hydrophobic surfaces. The values were determined with a goniometer (CAM200, KSV NIMA-Biolin Scientific, Finland) and a detailed method can be found in Ref. [16]. Contact angles of 8.7° ± 0.8 and 8.5° ± 0.7 were obtained for the hydrophilic DPI chips and QCM-D chips, respectively. Contact angles of 88.1° ± 2.5 and 88.9° ± 5.5 were obtained for the hydrophobic DPI and QCM-D chips, respectively. The surfaces are therefore considered as ‘super-hydrophilic’ and ‘hydrophobic’ as the contact angle of one type is less than 10° and of another type is approximately 90° [23].

### 2.2 DPI

An *Ana*light^®^ dual polarization interferometer (*Ana*light^®^ 4D, Farfield–Biolin Scientific AB, Sweden) was used to optically characterize adsorption of the three proteins on both hydrophilic and hydrophobic sensor chips. Details of the instrumentation can be found elsewhere [16,24]. The instrument alternately generates two orthogonal polarizations of light that excite waveguide modes supported by the DPI sensor chip. These two polarization waveguide modes are known as the transverse electric (TE) and transverse magnetic (TM) modes. The parameters employed in the experiments such as the operating temperature, flow rate, protein solution injection volume and the bulk solution exchange rate, together with the standard calibration procedure prior to the experiments, the cleaning procedure have been described in our previous work [16]. Successive protein solutions were injected until surface saturation was reached (as indicated by the TM and TE signals where further injections would not lead to further signal phase increment), then followed by a 30 min buffer rinsing. Data were analysed using *Ana*light explorer (Farfield–Biolin Scientific AB, Sweden) to calculate layer refractive index (RI), thickness, mass and density. During protein incubation, correction of the protein solution RI was performed, in order to obtain accurate values of the protein layer density and mass. Details of the new bulk RI calculations can be found in our previous work [16].

### 2.3 QCM-D

In parallel to DPI, QCM-D was also performed to record real time change of frequency and dissipation value during protein adsorption (QCM-D E4, Q-Sense-Biolin Scientific AB, Sweden). A detailed description of the instrument and the experiment design can be found in our previous work [16]. Alternating protein and buffer solutions of fixed volume were passed over the substrate until the surface was saturated. This was followed by 30 min of buffer rinsing.

For the adsorbed layer mass calculation, the Sauerbrey equation is employed in lysozyme adsorption on both surfaces as the average dissipation value is less than 1 × 10^−6^ (Eq. (1)). The Voigt model is used to calculate BSA and α-Syn adsorbed mass due to greater dissipation values.

(1) ΔM=−CΔf/n

Where Δ*M*, *C*, and *n* represent the adsorbed mass per unit area, mass sensitivity constant (17.7 ng cm^−2^ Hz^−1^), and the overtone number, respectively. The fifth overtone was used for analysis.

The Voigt model was also used to obtain the viscoelastic properties of all three adsorbed protein layers on both surfaces. The fixed parameters were bulk solution density and bulk solution viscosity, which were assumed as 1000 kg/m^3^ and 0.001 kg/ms, respectively. The parameters available to fit were the layer viscosity, layer shear modulus and layer thickness, which were set in the range of 0.0001–0.1 kg/ms, 1 × 10^4^ and 1 × 10^8^ Pa, and 1 × 10^−10^ and 1 × 10^−6^ m, respectively. Overtones *n* = 3, 5, 7, 9, 11, and 13 were employed for the modelling [16].

For the percentage layer solvation calculation, the ‘dry’ mass obtained from the DPI (Δ*M*_ads_) was subtracted from the QCM-D calculated ‘wet’ mass (Δ*M*_qcmd_), then divided by Δ*M*_qcmd_ (Eq. (2)).

(2) $$\text{wt}\%\;\text{solvation}=\left(\Delta M_{\text{qmcd}}-\Delta M_{\text{ads}}\right)/\Delta M_{\text{qmcd}}$$

## 3 Results

### 3.1 Quantification of protein layer solvation

**Fig. 1:**
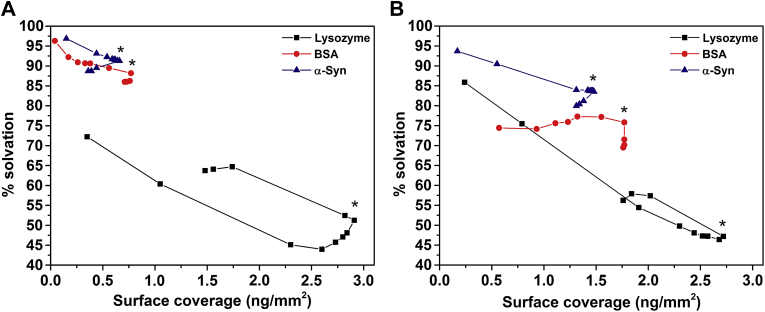
Evolution of the adsorbed layer hydration throughout the protein incubation and buffer rinsing process on hydrophilic surface ‘Silicon oxide’ (**A**) and hydrophobic surface ‘Methyl’ (**B**). * Indicates when the bulk solution was replaced by protein-free solution i.e. rinsing starts from that point. Data extracted from one experiment.

One important property obtained from combining the adsorbed mass values from both DPI and QCM-D is the protein layer solvation (wt% solvation). Quantification of the entrapped solvent and its evolution throughout the adsorption process are important factors that directly link to the performance of artificial materials [15,25,26]. The change of wt% solvation during the adsorption and desorption processes of lysozyme, BSA and α-Syn on both hydrophilic and hydrophobic surfaces are calculated using Eq. (2) and presented in Fig. 1A and Fig. 1B, respectively. As noted in Fig. 1, the wt% solvation decreases as the surface coverage increases for all the proteins adsorbed on both surfaces until surface saturation (indicated by the *). However, this is most significant for lysozyme, with starting values of 70% (Fig. 1A) or above (Fig. 1B), that drops to approximately 45%. In comparison, the changes of wt% solvation for BSA and α-Syn through the processes are much less significant.

Another interesting finding is related to the wt% layer hydration of different proteins at the same surface coverage, which follows a trend of α-Syn> BSA > lysozyme. Moreover, comparing the effect of the surface polarity on the layer hydration for a given protein, a higher hydration level is observed on the hydrophilic surface at surface saturation coverage (as indicated by the *) for BSA and α-Syn (91% on ‘Silicon oxide’ substrate compared to 83% on ‘Methyl’ substrate for α-Syn; 88% on ‘Silicon oxide’ substrate compared to 77% on Methyl substrate for BSA) whereas this effect is not significant for lysozyme (both adsorbed layers have approximately 50% solvation).

### 3.2 Other layer properties

#### 3.2.1 Adsorbed mass

**Fig. 2:**
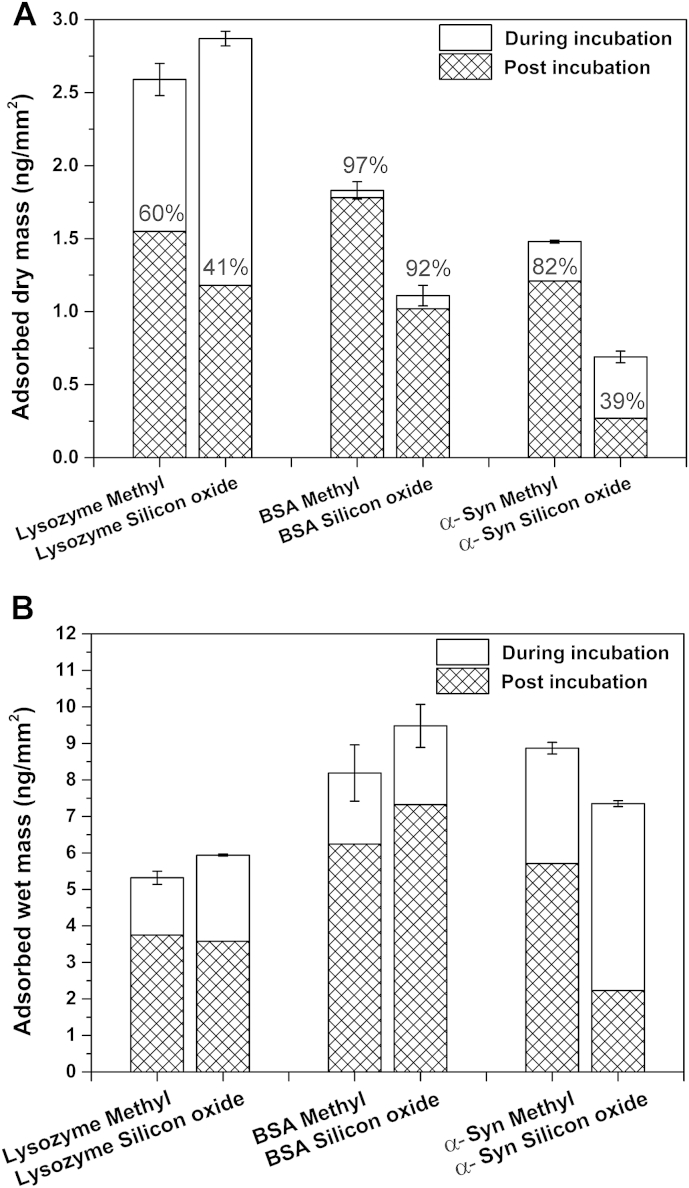
The adsorbed mass sensed by both DPI (**A**) and QCM-D (**B**) of all three proteins on both surfaces (where ‘Silicon oxide’ indicates hydrophilic surface and ‘Methyl’ indicates the hydrophobic surface) during incubation of protein (during incubation) and rinsing (post incubation). % Values represent the percentage of mass remaining post incubation of proteins. Data is expressed as mean ± SD (*n* = 3).

Apart from the wt% solvation, other useful layer properties can also be extracted from DPI and QCM-D data, one of which is the adsorbed protein mass detected by DPI (‘dry’ mass) and the adsorbed layer mass sensed by QCM-D (‘wet’ mass). The data presented in Fig. 2 show the variation in ‘dry’ mass and ‘wet’ mass obtained from lysozyme, BSA and α-Syn on both surfaces during and after protein incubation. A number of interesting features can be found. Firstly, as shown in Fig. 2A, lysozyme has the highest adsorbed ‘dry’ mass in a monolayer coverage range [16] on both surfaces during protein incubation (2.6 ± 0.1 ng/mm^2^ on the hydrophobic surface and 2.87 ± 0.05 ng/mm^2^ on the hydrophilic surface), followed by BSA (1.83 ± 0.06 ng/mm^2^ on the hydrophobic surface and 1.11 ± 0.07 ng/mm^2^ on the hydrophilic surface) and finally α-Syn that presents the lowest adsorbed ‘dry’ mass (1.48 ± 0.01 ng/mm^2^ on the hydrophobic surface and 0.69 ± 0.04 ng/mm^2^ on the hydrophilic surface). However, the QCM-D-sensed mass values of all proteins shown in Fig. 2B display the reversed trend: lysozyme has the lowest adsorbed layer mass (∼5.5–6 ng/mm^2^) detected during adsorption on both surfaces despite its highest ‘dry’ mass content; followed by BSA and α-Syn with similar adsorbed layer mass values (∼7.5–10 ng/mm^2^). This finding is attributed to the lower solvation of lysozyme layers.

Another interesting finding is the enhanced effect of the surface hydrophobicity on the adsorption of BSA (60% more protein adsorption on ‘Methyl’ substrate) and α-Syn (110% more protein adsorption on ‘Methyl’ substrate) as shown in Fig. 2A. Conversely, lysozyme adsorption is barely influenced by the change of surface polarity, producing the same surface coverage on both substrates. Furthermore, lysozyme exhibits a large extent of desorption on both surfaces, as only 60% of the surface saturation coverage (‘dry’ mass) remains on the hydrophobic substrate and 41% on the hydrophilic substrate after rinsing. Little desorption is observed for BSA on both substrates (more than 90% ‘dry’ mass remaining), whereas desorption is substrate-dependent in the case of α-Syn (82% of the ‘dry’ mass remains on ‘Methyl’ substrate and only 39% on ‘Silicon oxide’ substrate).

#### 3.2.2 Layer thickness

**Fig. 3:**
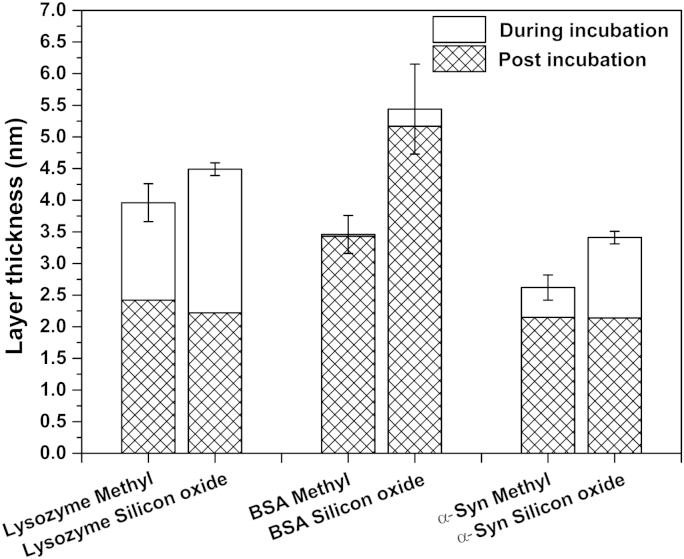
Layer thickness of all three proteins adsorbed on both surfaces (where ‘Silicon oxide’ indicates the hydrophilic surface and ‘Methyl’ indicates the hydrophobic surface) during incubation of protein (during incubation) and rinsing (post incubation). Data is expressed as mean ± SD (*n* = 3).

The thickness of the adsorbed protein layers on each surface during and post incubation of proteins is presented in Fig. 3. The layer thickness is an important parameter that indicates whether there is a change of the conformation of the adsorbed protein (e.g. proteins are deformed, unfolded or globular during adsorption). In addition, it also gives information about protein orientation within the layer (e.g. the direction in which proteins are adsorbed onto the surface). As shown in Fig. 3, the thickness of lysozyme layers adsorbed on both surfaces are in the range between 4 and 4.5 nm during adsorption. As the dimensions of lysozyme protein are 30 × 30 × 45 Å^3^ [27], these thickness values imply an end-on dominated orientation of lysozyme molecules on both surfaces. The thickness values on both substrates decrease to ∼ 2.5 nm post incubation of lysozyme suggesting a protein rearrangement upon rinsing as previously described [16]. The thickness of BSA layers greatly differs on different surfaces: it is 3.5 nm on the hydrophobic surface and 5.5 nm on the hydrophilic surface. These values remain similar post incubation of BSA suggesting no noticeable rearrangement in the layer structure. The thickness values obtained here are within the published range [28–30]. Concerning α-Syn, the layers formed during protein incubation display thickness within the range 2.6–3.5 nm, with the highest value on the hydrophilic surface. Similar to BSA, the thicker layer on the hydrophilic surface may indicate a different orientation of the adsorbed α-Syn compared with the hydrophobic surface, and will be discussed later. During rinsing, the thickness values reach a similar value on both surface of ∼2 nm suggesting the same conformation of residual proteins on both substrates.

#### 3.2.3 Layer stiffness and density properties

**Fig. 4:**
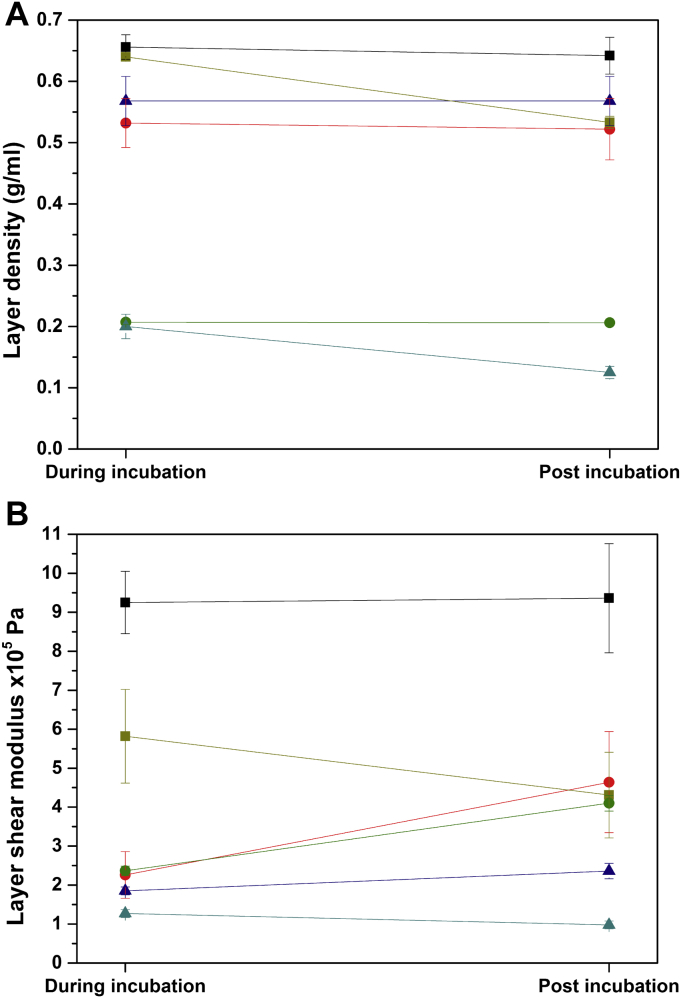
Layer density (**A**) and layer shear modulus (**B**) measured for all three proteins adsorbed onto different surfaces during incubation of protein (during incubation) and rinsing (post incubation). Different conditions are indicated by different colours: lysozyme on ‘Methyl’ (black) and ‘Silicon oxide’ (dark yellow), BSA on ‘Methyl’ (red) and ‘Silicon oxide’ (green), α-Syn on ‘Methyl’ (navy) and ‘Silicon oxide’ (cyan). Data is expressed as mean ± SD (*n* = 3). (For interpretation of the references to colour in this figure legend, the reader is referred to the web version of this article.)

Stiffness is used to describe the rigidity of a material, usually expressed as Young's modulus, the bulk modulus, or the shear modulus. In terms of protein adsorption, this property gives information of the compactness of the adsorbed layer. Density is also an indicator of the layer's compactness. From these parameters, the mechanical characteristic of the adsorbed protein layer can be studied. The layer density values calculated, during incubation of protein and rinsing, for all three proteins on both surfaces, are presented in Fig. 4A, and layer shear modulus values are shown in Fig. 4B. The layer density trend before and after rinsing from highest to lowest is as follows: lysozyme on ‘Methyl’/’Silicon oxide’ substrates >α-Syn/BSA on ‘Methyl’ substrate >α-Syn/BSA on ‘Silicon oxide’ substrate. The layer shear modulus trend from highest to lowest is: lysozyme on ‘Methyl’ substrate >lysozyme on ‘Silicon oxide’ substrate >α-Syn/BSA on ‘Methyl’/’Silicon oxide’ substrates. The highest density and shear modulus of the adsorbed lysozyme layers are in good agreement with the low water content previously shown in Fig. 1. Lysozyme layers are denser than the other two proteins. Another interesting finding is the difference in density of the BSA and α-Syn layers adsorbed onto different surface polarity (Fig. 4A). On average, the layer densities of both proteins adsorbed onto the hydrophobic surface (0.53–0.57 g/ml) are approximately 3 times those of the proteins adsorbed onto the hydrophilic surface (0.2 g/ml), suggesting a great influence of the surface polarity on the packing of the layer formed. The greater layer compactness obtained here is consistent with the lower wt% layer solvation (Fig. 1) and the lower layer thickness (Fig. 3) obtained on the hydrophobic surface for the two proteins. All of these data indicate the formation of a thick, diffuse layer on the hydrophilic surface, and a thin, dense layer on the hydrophobic surface. During rinsing, the layer density values of BSA remain similar on both substrates associated with an increase in shear modulus. For α-Syn, apart from a slight decrease of the density during rinsing, no noticeable change is observed.

## 4 Discussion

Protein adsorption at the liquid/solid interface is a field of focused research as this common phenomenon found in many biological processes depends strongly on the interplay between protein and surface [1]. Among the important questions arising in this field is the influence of protein properties on the formation and properties of the resulting layers. In order to correlate these variables, an accurate description of the layer properties must be used. However, a comparison of surface coverage alone is not sufficient in order to understand the diverse layer properties produced with the same surface coverage. This is because mass coverage value must be related to the water content and to the structural and viscoelastic properties of the layers in order to draw conclusions about the influence of protein and substrate properties from such data.

### 4.1 Interpretation of DPI and QCM-D data

The combination of the two-biosensing techniques, DPI and QCM-D, provides two different and complementary measurements of mass coverage [16]. DPI monitors the protein ‘dry’ mass using a direct measurement of thickness and RI, whereas QCM-D measures the protein mass and the water associated with the layer. Previously, we have proposed a model of lysozyme adsorption for which the layer properties were highly dependent on the surface coverage [16]. In the present study, we are investigating the adsorption process of lysozyme, on two substrates with different polarity, as a small, globular and rigid protein in comparison to α-Syn, an unfolded protein with a similar molecular weight (∼14 kDa) and BSA, a globular protein with a higher molecular weight (∼66 kDa).

Interestingly, the data presented here, show that the affinity for the substrates as monitored by mass coverage is different depending on the technique used. The mass coverage values measured by DPI follow the trend Lys > BSA > α-Syn whereas they follow the trend BSA∼α-Syn > Lys with QCM-D. This discrepancy is a result of the water content within the protein layers sensed by the QCM-D that is the sum of the trapped solvent in the interstices, the trapped solvent within the protein itself and finally the protein hydration layer [16]. α-Syn layers have the highest water on both substrates followed by BSA and finally lysozyme having the least water content within the adsorbed protein layer formed. This difference in layer solvation is a result of the size, shape and structure of proteins as well as their packing arrangement upon adsorption. Our data suggest that for the same surface coverage, α-Syn molecules have the largest intermolecular space for the water to occupy, compared to the bigger BSA molecules, and the small and compact lysozyme molecules. The amount of water trapped within a protein is a major contributor to the overall solvent content of the protein layer, and therefore for similar molecular weight, an unfolded protein overall forms a more hydrated layer.

### 4.2 Adsorption onto surfaces of opposite polarity

Lysozyme shows the highest affinity for the hydrophilic surface, followed by BSA and α-Syn. The dense packing arrangement might result from the favourable electrostatic attraction, promoted by the low ionic strength, between the positively charged protein (a net charge of +7 at pH 7.4) and negatively charged surface that dominates over the protein–protein electrostatic repulsions. This is not the case for BSA (a net charge of −14 at pH 7.4) and α-Syn (a net charge of −9 at pH 7.4) for which the electrostatic repulsion with the substrates and in between proteins prevents the formation of highly dense layers.

On the hydrophobic surface, a greater extent of protein adsorption is seen and the same trend is observed with lysozyme showing the highest adsorption followed by BSA and then α-Syn. The reduced thickness values measured on the hydrophobic surface for BSA and α-Syn suggest that these proteins undergo conformational reorientation or deformation leading to stronger protein-surface and inter-protein interactions.

### 4.3 Dependence of layer properties on the interplay between protein and surface

**Fig. 5:**
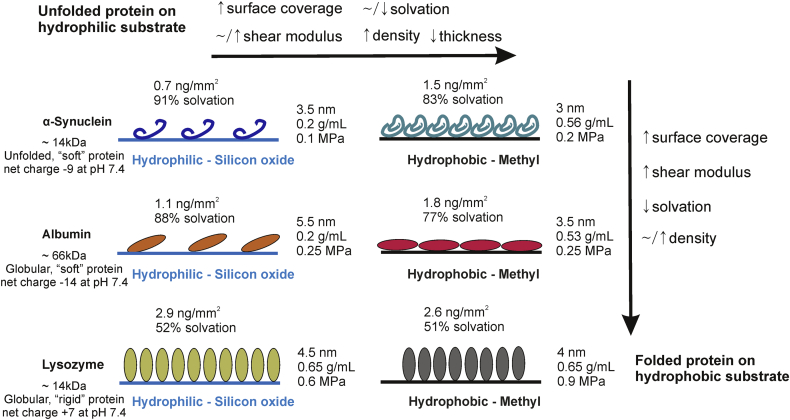
Surface coverage and layer properties of adsorbed proteins on the hydrophilic and hydrophobic substrates. Lysozyme and α-Syn share similar molecular weights but their affinity for the substrates and layer properties are contrasting due to their structural state. The properties of lysozyme layers are more sensitive to change in surface coverage. α-Syn and BSA change their orientation and packing arrangement on the hydrophobic surface inducing the formation of more compact and less hydrated layers.

The high affinity of lysozyme for both substrates is associated with the lowest water content, the highest shear modulus and highest density (Fig. 5). The thickness values of lysozyme layers indicate that proteins have adopted an end-on orientation on both surfaces, with a slightly lower value on the hydrophobic surface probably resulting from a slight deformation [16]. This lower thickness is associated with a higher shear modulus reflecting the formation of a more rigid layer. Apart from this change, lysozyme is not affected by the surface polarity as coverage and solvation values are similar. Upon rinsing, lysozyme adsorption exhibits a high level of reversibility on both substrates, with more desorption on the hydrophilic substrate. During the desorption process, as previously observed, a rearrangement of proteins to a side-on orientation occurs on both substrates resulting in layers with similar thickness values.

The large, unfolded proteins, on the other hand, are more sensitive in surface polarity than coverage as the increase in surface coverage does not significantly affect the water content for α-Syn and BSA layers.

This result can be related to the intrinsic stability of these proteins upon adsorption [31,32]. The “hard” protein lysozyme, which maintains its original conformation and undergoes limited structural changes, is not affected by the polarity of the surface. On the other hand, “soft” proteins such as α-Syn and BSA undergo conformational changes or deformations that are highly dependent on the surface polarity.

These changes appear to be the main reason for the decrease in water content. Indeed, a different packing arrangement of α-Syn on the hydrophobic substrate results in a significantly higher density and a slight increase in shear modulus. The density and thickness values show that α-Syn has adopted a more compact structure on the hydrophobic substrate. However, due to the unfolded nature of α-Syn a detailed description of protein orientation within the layers is limited. Greater adsorption and lower desorption are observed on the hydrophobic surface compared to the hydrophilic substrate resulting from α-Syn structural rearrangements. This behaviour contrasts with that of lysozyme that, as a rigid protein, shows the highest reversibility.

BSA layers show lower water content on the hydrophobic surface as the layer is thinner and more compact. Considering the dimensions of BSA in the globular state of 4 nm × 4 nm × 14 nm [33] (theoretical full monolayer coverage with proteins on a side-on orientation of 1.96 ng/mm^2^), the present coverage values show the formation of a sub-monolayer on the hydrophilic surface and a monolayer on the hydrophobic surface. The electrostatic repulsions might explain the lower adsorption on the hydrophilic surface and the arrangement of proteins on an edge-on orientation (Fig. 5). On the hydrophobic surface, the protein molecules adopt a side-on orientation to form a densely packed monolayer. These different orientations might be responsible for the lower percentage of solvation for the same surface coverage. Similarly to α-Syn, surface hydrophobicity leads to more BSA adsorption and less desorption. This change of the surface polarity enhances the contribution of hydrophobic interaction and hence promotes the adsorption.

Together, these results reveal that protein layer properties can be tuned by varying the structure, size and charge of the adsorbed proteins and the surface polarity (Fig. 5). Although lysozyme and α-Syn are similar in molecular weight, the layers formed on the hydrophilic silicon oxide surface are different; the α-Syn layer is diffuse and soft with a high percentage of solvation, whereas the lysozyme layer is highly packed, rigid and has low water content. Interestingly, for a similar molecular weight, the unfolded protein α-Syn forms a layer with the highest percentage of solvation, which is not a function of surface coverage but is due to the highest water content trapped within the protein. BSA, characterized by a higher molecular weight, shows some similarities in the layer properties with α-Syn forming a more hydrated, softer and less dense layer (compared to lysozyme). The surface polarity influences the resulting layer properties of α-Syn and albumin with more packed, rigid and less hydrated layers being formed on the hydrophobic methylated surface due to different orientation and packing of these proteins. By contrast, lysozyme is barely influenced by the change of surface polarity. These effects are related to the difference in the intrinsic structural stability of these proteins upon adsorption.

A trend in the layer properties can be drawn from the data extracted in this study showing that for a similar molecular weight a “soft”, unfolded protein adsorbed onto a hydrophilic surface with unfavourable protein-surface interactions presents lower density packing arrangement with higher water content and lower rigidity than a “rigid”, folded protein adsorbed onto a hydrophobic surface.

## 5 Conclusions

The combination of two biosensing techniques is used in the present study to obtain complementary information about the properties of protein layers adsorbed at the liquid/solid interface. This approach describes in detail the packing, orientation and structure of proteins, along with the water content and viscoelastic properties of the layers. We show that the layers formed are highly dependent on the interplay between protein and surface as solvation and packing arrangement can be different even for proteins sharing similar molecular weight. The surface polarity influences the resulting layer properties, where the extent to which depends on the intrinsic structural stability of the proteins upon adsorption. Importantly, the trend in the layer properties extracted from this study will help to make predictions of the adsorption process of other proteins and on the effect of the interplay between protein and surface on the properties of layers formed at the solid/liquid interface.
